# Edible and Medicinal Mushrooms as Sources of Bioactive Molecules in Pregnancy: Potential Impact on Preeclampsia and Gestational Diabetes Outcomes

**DOI:** 10.3390/molecules31132355

**Published:** 2026-07-03

**Authors:** Dragan Stajić, Mirjana Bogavac, Marko Stojić, Gabriel Stefan Nađ, Marko Ilinčić, Maja Karaman, Milena Rašeta, Jovana Mišković

**Affiliations:** 1 Department of Obstetrics and Gynecology, Faculty of Medicine, University of Novi Sad, 21000 Novi Sad, Serbia; 2 Department of Obstetrics and Gynecology, University Clinical Centre of Vojvodina, 21000 Novi Sad, Serbia; 3 ProFungi Laboratory, Department of Biology and Ecology, Faculty of Sciences, University of Novi Sad, Trg Dositeja Obradovića 2, 21000 Novi Sad, Serbia; 4 Department of Chemistry, Biochemistry and Environmental Protection, Faculty of Sciences, University of Novi Sad, Trg Dositeja Obradovića 3, 21000 Novi Sad, Serbia

**Keywords:** edible mushrooms, medicinal mushrooms, ergothioneine, antioxidants, oxidative stress, pregnancy, preeclampsia, gestational diabetes mellitus, maternal health, functional foods

## Abstract

Pregnancy involves profound metabolic, hormonal, and immunological adaptations essential for fetal development; however, disturbances may lead to complications such as preeclampsia (PE), pre-gestational diabetes, and gestational diabetes mellitus (GDM). These conditions are closely linked to oxidative stress, inflammation, endothelial dysfunction, impaired placentation, and metabolic dysregulation. Consequently, dietary strategies capable of modulating these pathways are of increasing interest. Edible and medicinal mushrooms are widely studied as functional food due to the content of bioactive compounds with antioxidant, anti-inflammatory, immunomodulatory, and metabolic regulatory effects. This review summarizes the nutritional composition of mushrooms and highlights key bioactive constituents with antioxidant and metabolic regulatory properties. Among them, ergothioneine has emerged as a key molecule due to its potent redox-buffering capacity and its potential involvement in the activation of the Nrf2 signaling pathway, a master regulator of cellular antioxidant defense. Through modulation of Nrf2-dependent gene expression, mushroom-derived compounds may contribute to improved cellular resilience against oxidative damage relevant to PE and GDM pathophysiology. Mushroom consumption has additionally been associated with improved glycemic control and enhanced antioxidant defenses in experimental and limited clinical studies, although evidence regarding the prevention or management of hypertensive and metabolic pregnancy complications remains insufficient. Although preclinical findings are promising, clinical evidence remains limited. Further well-designed prospective studies and randomized controlled trials are required to determine efficacy, safety, optimal intake, and active compounds responsible for these effects. Nevertheless, current evidence supports the biological plausibility that edible and medicinal mushrooms are promising dietary modulators of the ergothioneine–Nrf2 axis with potential relevance for maternal–fetal health.

## 1. Introduction

Preeclampsia (PE) and gestational diabetes mellitus (GDM) are among the most prevalent and severe complications of pregnancy, significantly contributing to maternal and perinatal morbidity and long-term metabolic consequences for both mother and offspring [1,2,3]. According to the World Health Organization (WHO), PE affects approximately 3–8% of pregnancies worldwide and remains a leading cause of maternal and perinatal morbidity and mortality, accounting for nearly 16% of maternal deaths globally [4,5]. Key risk factors include nulliparity, obesity, multiple pregnancy, chronic hypertension, diabetes, and kidney disease, while in parallel, diabetes has emerged as one of the fastest-growing global public health challenges [6]. Reflecting these global trends, the prevalence of pre-gestational diabetes among pregnant women has increased substantially over the past decade in Belgrade, Serbia, and is projected to continue rising, potentially doubling by 2050 [7]. Although clinically distinct, PE and GDM share several pathophysiological mechanisms, including oxidative stress, chronic low-grade inflammation, endothelial dysfunction, impaired glucose homeostasis, and abnormal placental development, many of which originate during early pregnancy [2,3].

Early gestation represents a critical window during which disturbances in redox balance, immune adaptation, and metabolic regulation may predispose women to the later development of PE and GDM [8]. Over recent decades, socioeconomic development, urbanization, and globalization have profoundly altered traditional dietary patterns in many Western countries, promoting increased consumption of highly processed foods rich in refined sugars and saturated fats. Although balanced nutrition is widely recognized as a cornerstone of a healthy lifestyle, excessive intake of energy-dense foods remains common and contributes to metabolic dysregulation, oxidative stress, chronic low-grade inflammation, and cardiovascular dysfunction [9], all of which are established risk factors for pregnancy-related and other non-communicable diseases [10]. Nutrition is increasingly acknowledged as one of the most important environmental factors influencing immune system development and function [11]. Consequently, diets rich in fruits, vegetables, and other natural sources of bioactive compounds are considered particularly beneficial for maintaining health and reducing disease risk [12]. Among these compounds, naturally occurring antioxidants have attracted considerable attention as bioactive compounds due to their potential to provide physiological benefits beyond basic nutritional requirements. In line with this concept, nutritional interventions are increasingly being investigated as preventive strategies for PE and GDM [13,14,15]. However, while most studies have focused on conventional micronutrients and dietary patterns, biologically rich food sources such as edible and medicinal mushrooms remain largely overlooked despite their abundance of antioxidant, anti-inflammatory, immunomodulatory, and metabolically active compounds [16]. At the same time, there is growing interest in identifying safe, food-based preventive strategies that can complement existing prenatal care, particularly for pregnancy complications in which pharmacological options remain limited. Clarifying the potential role of mushrooms within this framework is therefore of considerable scientific and clinical relevance, as it may provide new insights into nutritional approaches for improving maternal and fetal health outcomes.

While the exact mechanisms that promote GDM are poorly understood, GDM is associated with inadequate functional β-cell mass expansion and with a systematic increase in oxidative stress. During late pregnancy, physiological insulin resistance increases to meet fetal glucose demands, requiring adaptive expansion of maternal pancreatic β-cell mass to maintain normal glucose levels [17]. Failure of this adaptive response can contribute to GDM, which is associated with reduced β-cell function and increased oxidative stress. Haidery et al. [18] demonstrated in a preclinical mouse model that nuclear factor erythroid 2-related factor 2 (Nrf2) is upregulated in pancreatic β-cells during pregnancy and is essential for maintaining β-cell proliferation, survival, and redox balance. β-Cell-specific deletion of Nrf2 leads to increased oxidative damage, impaired β-cell expansion, and disrupted glucose homeostasis in late gestation [19]. Furthermore, 17β-estradiol enhances Nrf2 signaling and protects β-cells against oxidative stress, suggesting a key hormone–redox interaction. Overall, Nrf2 plays a central role in β-cell adaptation during pregnancy and represents a potential therapeutic target for GDM.

Identifying these risks early opens the possibility for exploring natural Nrf2 activators, such as mushroom-derived polyphenols, as potential supportive strategies to mitigate oxidative stress-induced endothelial damage.

Mushrooms (fungi) represent a unique class of functional foods with a distinctive nutritional and metabolic profile, characterized by high levels of dietary fiber, essential micronutrients, and a wide spectrum of bioactive compounds, including β-glucans, ergothioneine, and various phenolic metabolites [20]. These compounds have been shown to exert antioxidant, anti-inflammatory, and immunomodulatory effects [16,20,21,22,23,24,25,26,27,28], which are mechanistically relevant to key processes implicated in the pathogenesis of PE and GDM, particularly oxidative stress, endothelial dysfunction, and chronic low-grade inflammation [1,2,3]. Despite this biological plausibility, the current body of evidence linking mushroom-derived metabolites with pregnancy-related metabolic and vascular outcomes remains fragmented and has not yet been systematically synthesized.

Edible and medicinal mushrooms represent a potent source of bioactive metabolites capable of modulating these pathological pathways. Their high antioxidant capacity is crucial for neutralizing reactive oxygen species (ROS) and stabilizing the redox environment in the placenta. Their well-proven antioxidant activity is largely attributed to their richness in phenolic compounds and polysaccharides [16,20,21,22,23,24,25,26,27,28], which activate endogenous defense systems such as the Nrf2/ARE signaling pathway [29,30], enhancing cellular resistance to oxidative stress. Emerging evidence further indicates that mushroom-derived secondary metabolites, particularly triterpenoids, may contribute to these effects by promoting Nrf2-mediated antioxidant responses and reducing ROS-induced cellular damage [31]. Collectively, these findings support the potential of edible mushrooms as natural regulators of redox balance, which is frequently disrupted in both PE and GDM.

Clinical trials with antioxidant interventions in PE have not proved successful. Based on the current literature, enhancement of the endogenous protection system against oxidative stress by Nrf2 activation seems to be a promising alternative. Thus, supplementation with Nrf2 inducers in a therapeutic design could be an innovative target that deserves further intensive research in well-conducted studies [19].

Mushroom consumption within self-selected diets is generally associated with favorable cardiometabolic effects [32]. A recent narrative review concluded that whole mushroom consumption consistently reduces serum triglycerides, while exhibiting neutral effects on HDL, LDL, and C-reactive protein concentrations, with limited evidence also suggesting beneficial effects on blood pressure and immune function through increased salivary immunoglobulin A levels [33]. Furthermore, when incorporated into healthy dietary patterns, *Agaricus bisporus* and *Pleurotus ostreatus* have been shown to improve fasting serum glucose concentrations, indicating a potential role in supporting glucose homeostasis [34]. Although evidence for several cardiometabolic biomarkers remains limited or inconsistent, no adverse effects of mushroom consumption have been reported across intervention studies [8,13,14,15]. Given that impaired glycemic control, dyslipidemia, inflammation, and endothelial dysfunction are key features of GDM and PE, these findings support further investigation of edible mushrooms as dietary components for improving maternal cardiometabolic health.

Supporting these clinical observations, experimental studies with *Ganoderma* species have demonstrated antioxidant activity, mitigation of diabetes-associated dyslipidemia, and potential hepato- and nephroprotective effects in diabetic animal models [35], suggesting that mushroom-derived bioactive compounds may contribute to the regulation of metabolic disturbances associated with hyperglycemia and oxidative stress.

This review therefore aims to critically evaluate and integrate the existing experimental and clinical evidence on mushroom consumption during pregnancy, with a particular focus on their mechanistic roles during early pregnancy and their potential implications for the prevention or modulation of PE and GDM. Special emphasis is placed on bridging data from in vivo and human studies, while also identifying key gaps in knowledge, methodological limitations, and challenges in translation toward clinical practice.

## 2. Pathophysiology of Preeclampsia and Gestational Diabetes: Targets for Early Nutritional Intervention

**Figure 1:**
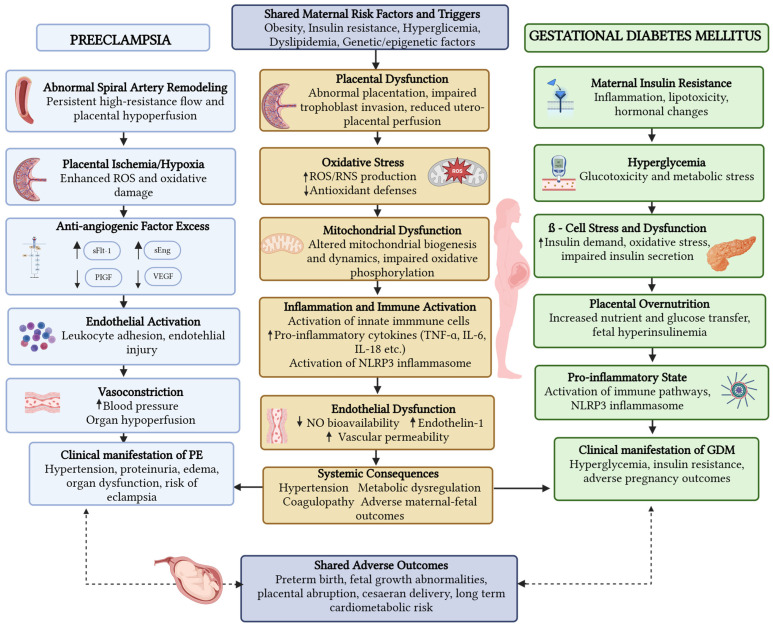
Common pathophysiology of preeclampsia and gestational diabetes mellitus (Created with BioRender.com; account holder: P. Davidovic; document ID: hg4w67r; https://BioRender.com).

PE and GDM are multifactorial pregnancy complications that share a common pathogenic framework characterized by impaired placentation, chronic low-grade inflammation, and severe metabolic imbalance (Figure 1). Both disorders originate in early gestation, often before clinical manifestations arise, underscoring the necessity for early preventive interventions.

PE represents a multisystem hypertensive disorder of pregnancy characterized by new-onset hypertension (≥140/90 mmHg) after 20 weeks of gestation, primarily driven by defective extravillous trophoblast invasion and inadequate remodeling of uterine spiral arteries. This leads to placental hypoxia-reoxygenation injury, which activates oxidative pathways (e.g., xanthine oxidase and mitochondrial dysfunction), resulting in excessive ROS generation and maternal endothelial distress [36]. Characterized by defective placentation and systemic endothelial dysfunction, PE remains a major contributor to adverse pregnancy outcomes worldwide. Utero-placental development in pregnancy depends on direct maternal-fetal interaction in the uterine wall decidua. Abnormal uterine vascular remodeling preceding placental oxidative stress and placental dysfunction are associated with PE and fetal growth restriction [37].

Effective management necessitates early risk identification; thus, 1st-trimester screening has emerged as a cornerstone of modern prenatal care. Current strategies emphasize a multimodal approach, combining maternal history with specific biomarkers to enhance risk prediction and clinical decision-making during this critical early window [38]. Nrf2 can be regulated by important cell modulators such as DJ-1, VEGF, oxLDL, LXA4, AOPPs, miR-133a-3p, CD151 and BRD4, favoring the cell response to oxidative stress, avoiding apoptosis and ferroptosis and promoting cell proliferation. Natural and synthetic compounds (e.g., melatonin, resveratrol, apocyanin) can act as potent antioxidants, activating the Nrf2/KEAP1 signaling pathway, reducing apoptosis and inflammation [39] and probably improving the clinical signs of PE.

GDM is characterized by progressive insulin resistance and pancreatic β-cell failure. Maternal hyperglycemia promotes mitochondrial dysfunction and ferroptosis, an iron-dependent form of regulated cell death, within placental tissues, further impairing insulin signaling [40].

Pregestational diabetes, characterized by pre-existing insulin resistance or absolute insulin deficiency, creates a pro-inflammatory environment that exacerbates systemic oxidative stress and vascular complications during pregnancy [41]. Hence, mushroom-derived triterpenes and polyphenols, such as those found in *Ganoderma lucidum*, may mitigate these complications by activating the Nrf2 pathway, thereby enhancing the cellular antioxidant defense and protecting pancreatic beta-cells from oxidative damage [42,43]. In addition to β-glucans, ergothioneine, and phenolic compounds, several edible and medicinal mushrooms have been reported to contain melatonin, an indoleamine with potent antioxidant and anti-inflammatory properties [44]. Given the established role of oxidative stress in the pathogenesis of PE and GDM, mushroom-derived melatonin may represent an additional bioactive component contributing to the health-promoting effects of mushrooms, although its specific role in pregnancy outcomes remains largely unexplored.

GDM arises from an insufficient maternal pancreatic response to the physiological insulin resistance of pregnancy, often linked to placental-derived hormones and chronic low-grade inflammation [45]. Thus, bioactive polysaccharides, particularly beta-glucans like schizophyllan, have shown potential in modulating glucose metabolism and improving insulin sensitivity through Nrf2 signaling [46].

In general, both PE and diabetes share a common pathophysiological thread of endothelial dysfunction and systemic inflammation, where the Nrf2 pathway serves as a critical regulatory node. This is the core point where the inclusion of unique mushroom compounds, such as ergothioneine, into the maternal diet could represent a novel strategy for reducing ROS levels and improving vascular health in high-risk pregnancies complicated by glucose intolerance.

## 3. Mushrooms as Functional Foods: The Role of Antioxidants in Pregnancy

Edible mushrooms represent valuable dietary sources of essential nutrients and biologically active compounds that may contribute to maternal and fetal health during pregnancy. Due to their low fat content, favorable amino acid composition, and abundance of micronutrients and antioxidant molecules [20,21,22,23,47,48], mushrooms have attracted increasing attention as functional foods with potential roles in the prevention of metabolic and inflammatory pregnancy-related disorders [49]. This interest is particularly relevant in the context of pregnancy complications such as PE and GDM, conditions in which oxidative stress, chronic low-grade inflammation, endothelial dysfunction, and impaired metabolic homeostasis are recognized as major pathogenic mechanisms [2,3].

Edible and medicinal mushrooms are characterized by a balanced macronutrient composition, including high-quality proteins, dietary fibers, and low levels of lipids [48,50]. Mushroom proteins contain essential amino acids important for maternal nutrition and fetal development, while their low caloric value makes them suitable components of balanced pregnancy diets. Particular attention has been given to mushroom-derived polysaccharides and dietary fibers, especially β-glucans, due to their potential effects on glucose metabolism, satiety, immune modulation, and gut microbiota regulation [48,51,52]. These properties may be especially beneficial during early pregnancy, when metabolic adaptations influence maternal insulin sensitivity and may contribute to the development of GDM in susceptible individuals. Furthermore, emerging evidence suggests that gut microbiota dysbiosis and altered immune responses are involved in both GDM and PE pathogenesis, highlighting the potential value of mushroom-derived dietary fibers as modulators of maternal metabolic and inflammatory status [53,54].

Mushrooms are natural sources of several micronutrients important during pregnancy, including selenium, potassium, B-complex vitamins, and vitamin D_2_ [55]. Selenium contributes to antioxidant defense systems [56] and may play a role in reducing oxidative stress associated with pregnancy complications. B vitamins participate in energy metabolism and fetal neural development, whereas potassium contributes to cardiovascular and electrolyte balance [57]. In addition, UV-exposed mushrooms can provide significant amounts of vitamin D_2_ [58,59], which is increasingly recognized as important for maternal immune regulation and fetal skeletal development [57]. Vitamin D insufficiency has also been associated with an increased risk of both PE and GDM, suggesting that vitamin D_2_-enriched mushrooms may represent a valuable dietary source of this nutrient during pregnancy. Moreover, selenium-dependent antioxidant enzymes, including glutathione peroxidases, may play important roles in protecting placental tissues from oxidative damage, a hallmark of PE development [56].

Beyond their nutritional value, mushrooms contain numerous bioactive molecules with antioxidant, anti-inflammatory, immunomodulatory, and metabolic activities [16,60,61,62]. Among the most extensively studied compounds are β-glucans, ergothioneine, phenolic compounds, and terpenoids [63,64,65,66]. β-Glucans are known for their immunomodulatory and metabolic regulatory effects [67,68], while ergothioneine acts as a potent antioxidant and cytoprotective molecule [65,69,70]. Phenolic compounds contribute to the antioxidant capacity of mushrooms through free radical scavenging mechanisms [24,25,26,27], whereas terpenoids, particularly abundant in medicinal mushroom species, exhibit anti-inflammatory and bioactive properties [71,72] that may be relevant in the context of pregnancy-associated metabolic disorders.

The antioxidant properties of these compounds are of particular interest because excessive production of ROS and insufficient antioxidant defenses contribute significantly to placental dysfunction, endothelial injury, and insulin resistance during pregnancy. Ergothioneine, a unique sulfur-containing antioxidant accumulated in mammalian tissues through a specific transporter (OCTN1), has attracted growing attention due to its ability to protect cells against oxidative and inflammatory damage [73]. Likewise, mushroom-derived phenolic compounds and terpenoids may support redox homeostasis by scavenging free radicals, modulating antioxidant enzymes, and regulating signaling pathways associated with inflammation and oxidative stress.

Although direct clinical evidence regarding mushroom consumption during pregnancy remains limited, experimental and epidemiological studies support the hypothesis that regular intake of edible mushrooms and their bioactive constituents may contribute to the maintenance of metabolic and vascular health. Through their combined antioxidant, anti-inflammatory, immunomodulatory, and glucose-regulating activities, mushrooms may represent promising functional foods for reducing risk factors associated with PE and GDM. Consequently, understanding the biological activities of mushroom-derived compounds may provide valuable insights into the development of nutritional strategies aimed at improving maternal and fetal outcomes during early pregnancy.

A comparative overview of the principal nutrients and bioactive compounds identified in commonly consumed edible mushrooms relevant to pregnancy health is presented in Table 1.

Before discussing the molecular mechanisms underlying the biological activities of mushroom-derived metabolites, it is important to consider their bioavailability and potential maternal-fetal disposition during pregnancy. Although the biological activities of ergothioneine and mushroom-derived β-glucans have been extensively investigated [49,65,70,73,76,78], considerably less is known about their pharmacokinetic behavior during pregnancy. Available evidence suggests that ergothioneine is efficiently absorbed via the organic cation transporter OCTN1 (SLC22A4) and accumulates in tissues exposed to oxidative stress [95]. However, data regarding its placental transfer, fetal distribution, and metabolism during human gestation remain limited. Likewise, β-glucans are believed to exert most of their biological effects through interactions with intestinal and immune cells rather than through extensive systemic absorption [52,68], and evidence for their transplacental passage is currently lacking. Therefore, although both compounds exhibit promising antioxidant and immunomodulatory properties, additional studies addressing their bioavailability, placental transport, metabolism, and fetal exposure are needed before their clinical application during pregnancy can be fully established.

## 4. Mushroom-Derived Bioactive Metabolites and Relevant Mechanisms

### 4.1. Antioxidant and Redox-Modulating Effects

**Figure 2:**
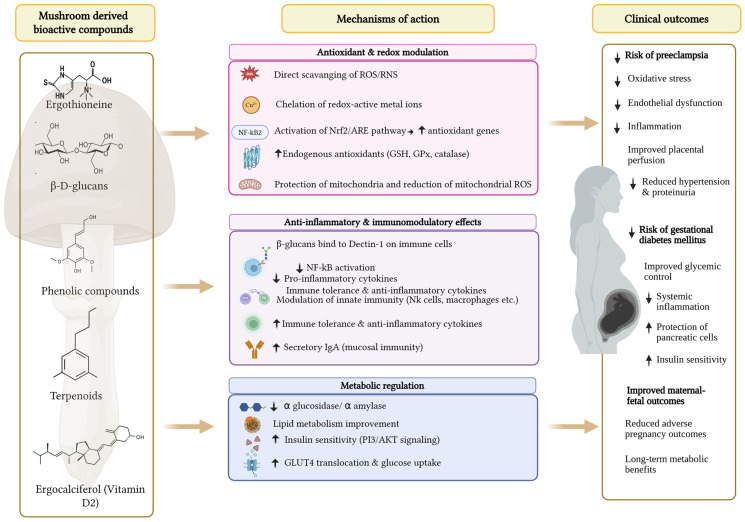
Potential mechanisms in the prevention of preeclampsia and gestational diabetes mellitus (Created in BioRender. Davidovic, P. (2026) aw2jzaj https://BioRender.com/).

Oxidative stress and inflammation are central contributors to the pathophysiology of both PE and GDM (Figure 2), particularly during early placental development [8]. Excessive production of ROS within the placenta disrupts redox homeostasis, impairs fetal growth, and alters immune regulation, thereby contributing to pregnancy complications such as PE and intrauterine growth restriction [96]. Given the critical role of redox balance in maintaining placental function, antioxidant systems are essential regulators of cellular signaling pathways involved in pregnancy adaptation [97]. In this context, mushroom-derived bioactive metabolites have gained attention due to their combined antioxidant, anti-inflammatory, and immunomodulatory properties.

Among these metabolites, ergothioneine, a sulfur-containing amino acid highly abundant in edible mushrooms [98], has emerged as a key compound of interest. Ergothioneine functions as a potent scavenger of ROS and nitrogen species and has been shown to protect cellular and mitochondrial integrity, while also exhibiting anti-inflammatory and cytoprotective effects [99]. Additional biological activities, including protection against UV-induced damage [100] and neuroprotection in models of neuronal injury [101,102], further support its broad redox-modulating capacity, although much of this evidence remains preclinical.

At the molecular level, L-ergothioneine exerts antioxidant effects through its thiol/thione tautomeric system, enabling efficient scavenging of reactive species and chelation of redox-active metal ions such as Cu^2+^ [103]. It has also been shown to activate the Nrf2/ARE pathway, thereby inducing endogenous cytoprotective antioxidant defenses. Importantly, its oxidized form can be readily recycled back to the reduced state by cellular thiols such as glutathione, ensuring sustained antioxidant capacity under physiological conditions [103,104].

From a physiological perspective, ergothioneine is efficiently absorbed in humans following dietary intake. Consumption of *A. bisporus* mushrooms increases circulating ergothioneine levels, confirming its bioavailability [78]. Moreover, oral administration of pure ergothioneine leads to rapid systemic uptake, long-term retention, and minimal urinary excretion, indicating the presence of active transport and conservation mechanisms in humans [105]. This is supported by the existence of a specific transporter, SLC22A4 (OCTN1), which facilitates selective tissue accumulation, particularly in organs exposed to oxidative stress [106].

The relevance of ergothioneine to pregnancy complications becomes more evident in the context of placental redox imbalance and mitochondrial dysfunction. Both PE and GDM are characterized by excessive oxidative stress, endothelial dysfunction, and impaired mitochondrial function [1,2]. In PE, mitochondrial ROS production contributes to defective placentation and maternal vascular injury [107], while in GDM, mitochondrial dysfunction is increasingly recognized as a key driver of inflammatory activation within the placenta [40].

A direct mechanistic link between ergothioneine and GDM-associated placental inflammation has been demonstrated in human tissue. McElwain et al. [8] showed that placentas from women with GDM exhibit increased mitochondrial oxidative stress, enhanced activation of the NLRP3 inflammasome, and elevated IL-18 production. Importantly, treatment of placental explants with L-ergothioneine significantly reduced IL-18 levels, indicating suppression of mitochondria-driven inflammasome activation. These findings suggest that ergothioneine may modulate a central inflammatory axis linking mitochondrial dysfunction to placental immune activation in GDM.

In addition to this direct evidence in GDM, experimental and epidemiological findings support a broader role for mushroom-derived antioxidants in metabolic regulation during pregnancy (Figure 2). For example, dietary supplementation with *L. edodes* has been shown to partially restore fetal insulin levels in experimental models of maternal diabetes, even in the absence of normalization of maternal glycemia [108]. Hyperglycemia-induced oxidative stress contributes to β-cell dysfunction, lipid peroxidation, and DNA damage, while also disrupting endogenous antioxidant defenses such as catalase and glutathione peroxidase [108,109,110]. Mushroom supplementation has been associated with improvements in antioxidant enzyme activity and reductions in lipid peroxidation markers, including TBARS, suggesting systemic redox stabilization [108]. These effects are consistent with findings from other medicinal mushrooms such as *Agaricus blazei* and *G. lucidum*, which also improve antioxidant defenses in diabetic models [111].

Supporting these mechanistic observations, epidemiological data indicate that dietary intake of white button mushrooms during early pregnancy is associated with a reduced incidence of gestational hypertension and PE, alongside improvements in systemic antioxidant biomarkers [32,112]. Regular consumption has also been linked to increased circulating ergothioneine levels and decreased markers of oxidative stress and inflammation [32]. These findings suggest that dietary mushrooms may exert systemic protective effects that are at least partially mediated by ergothioneine accumulation and redox modulation.

Finally, ergothioneine is uniquely positioned at the intersection of oxidative stress regulation, mitochondrial protection, and immune modulation. Its preferential uptake via SLC22A4 (OCTN1) [106] allows targeted accumulation in tissues experiencing oxidative stress, enabling modulation of ROS-dependent signaling pathways relevant to both PE and GDM. In particular, its ability to attenuate mitochondrial dysfunction-driven NLRP3 inflammasome activation provides a mechanistic framework linking dietary mushroom intake to reduced placental inflammation and improved metabolic adaptation during pregnancy [113].

Collectively, these data support a biologically plausible model in which mushroom-derived ergothioneine contributes to the regulation of oxidative stress, mitochondrial function, and inflammatory signaling pathways implicated in PE and GDM. While human bioavailability and mechanistic studies provide strong supportive evidence, definitive clinical trials in pregnant populations remain lacking. Therefore, ergothioneine should currently be considered a promising mechanistic candidate rather than a confirmed preventive agent for pregnancy-related metabolic and hypertensive disorders.

### 4.2. Anti-Inflammatory and Immunomodulatory Effects

In addition to redox regulation, mushroom-derived bioactive compounds exert important anti-inflammatory and immunomodulatory effects that are highly relevant to the pathophysiology of GDM and hypertensive pregnancy disorders. Chronic low-grade inflammation is a hallmark of both PE and GDM, closely intertwined with oxidative stress and metabolic dysregulation, and contributes to impaired placental function and altered maternal-fetal immune tolerance.

A major group of immunomodulatory compounds in mushrooms, such as polysaccharides, particularly β-D-glucans, influences both innate and adaptive immune responses. These molecules suppress NF-κB activation, reduce oxidative stress, and protect pancreatic β-cells from apoptosis and glucotoxicity, thereby supporting insulin secretion and improving glycemic control [114,115]. In parallel, β-glucans modulate insulin-related signaling pathways, including PI3K/AKT and GLUT4 translocation, contributing to improved insulin sensitivity and attenuation of inflammation-induced metabolic dysfunction [115,116].

Beyond polysaccharides, terpenoids and dietary fibers present in mushrooms further contribute to metabolic and immune regulation. Terpenoids inhibit α-glucosidase and α-amylase, thereby reducing postprandial glucose excursions that drive oxidative and inflammatory stress [117]. Dietary fiber delays glucose absorption and stabilizes glycemic variability, indirectly reducing inflammatory activation during pregnancy [115,117]. Mushrooms also represent a notable dietary source of vitamin D_2_, which may contribute to immunomodulation by influencing macrophage polarization and T-cell responses, although its role in GDM remains incompletely defined [118,119].

At the level of innate immunity, mushroom-derived β-glucans interact with pattern-recognition receptors such as dectin-1, triggering controlled activation of immune signaling cascades. This leads to regulated cytokine and chemokine production, enhanced phagocytosis, and improved immune surveillance while preventing excessive inflammatory responses [120,121]. β-glucans also promote dendritic cell maturation and antigen presentation, thereby supporting adaptive immune responses and maintaining immune homeostasis during pregnancy. Fungal β-glucans have been shown to exert stronger immunostimulatory effects compared to those derived from plant sources, further emphasizing their biological relevance [121].

In the context of pregnancy complications, immune dysregulation plays a central role in placental dysfunction. Altered macrophage activation and cytokine imbalance contribute to impaired vascular remodeling and increased inflammatory burden in both PE and GDM. Experimental evidence supports the ability of mushroom-derived compounds to modulate these processes. For instance, medicinal mushrooms such as *A. brasiliensis* and *G. lucidum* have been shown to normalize glycemic parameters, restore leukocyte homeostasis, regulate apoptosis-related pathways (p53/Bcl-2), and preserve erythrocyte membrane integrity in diabetic models, reflecting systemic anti-inflammatory and cytoprotective effects [122].

Additional support comes from studies on specific fungal metabolites. Cordycepin has been shown to reduce placental inflammation, modulate macrophage polarization, and improve vascular and fetal outcomes in experimental models of PE-like conditions [123]. These findings suggest that individual mushroom-derived compounds may act on distinct but interconnected inflammatory pathways involved in placental dysfunction.

Furthermore, experimental models of GDM indicate that mushroom supplementation can positively influence both maternal and fetal metabolic outcomes. Administration of *L. edodes* has been shown to partially restore fetal insulin levels in amniotic fluid, even in the absence of full normalization of maternal hyperglycemia, suggesting direct effects on fetal pancreatic function [108]. In diabetic pregnancy models, mushroom supplementation has been associated with increased catalase activity, improved glutathione peroxidase trends, and reduced lipid peroxidation, indicating systemic attenuation of oxidative-inflammatory stress [108]. Similar antioxidant and metabolic benefits have been reported for *Agaricus blazei* and *G. lucidum* in diabetic models [108,111].

Collectively, these findings support a multi-level immunometabolic model in which mushroom-derived compounds regulate immune cell activation, cytokine signaling, and metabolic stress responses. Through coordinated effects on innate immunity, inflammatory signaling pathways, and glucose metabolism, these bioactive molecules may contribute to the mitigation of pathological processes underlying GDM and hypertensive pregnancy disorders. However, despite strong mechanistic evidence from preclinical studies, well-controlled clinical trials in pregnant populations are still required to confirm these effects and define their clinical relevance.

### 4.3. Nrf2 as a Common Therapeutic Target in Pregnancy-Related Disorders

Oxidative stress and chronic inflammation are shared pathological features of pregnancy-related disorders, including preeclampsia, gestational diabetes mellitus, placental dysfunction, and fetal growth restriction [124]. Consequently, molecular pathways capable of simultaneously regulating cellular redox balance and inflammatory responses have emerged as attractive therapeutic targets. Among these, the Nrf2 signaling pathway has received considerable attention because of its central role in coordinating endogenous antioxidant defense mechanisms [125].

The studies summarized throughout this review consistently indicate that mushroom-derived bioactive compounds, particularly ergothioneine, β-glucans, polysaccharides, phenolic compounds, and triterpenoids, exert many of their beneficial effects through activation of the Nrf2/ARE pathway [126]. Nrf2 activation promotes the expression of cytoprotective enzymes such as HO-1, NQO1, SOD, CAT, GPx, and phase II detoxifying enzymes, thereby restoring redox homeostasis, reducing ROS accumulation, preserving mitochondrial function, and attenuating inflammatory signaling [127]. Although the individual studies focus on different experimental models, including placental trophoblasts, pancreatic β-cells, endothelial cells, and animal models of preeclampsia or gestational diabetes, the underlying mechanism appears remarkably consistent. Activation of Nrf2 is frequently accompanied by suppression of NF-κB-mediated inflammation, reduced production of pro-inflammatory cytokines, improved antioxidant capacity, and preservation of cellular viability and function [128].

Collectively, the available evidence identifies Nrf2 as a common molecular hub through which mushroom-derived bioactive compounds may simultaneously modulate oxidative stress, inflammation, and metabolic homeostasis during pregnancy. This integrated mechanism provides a plausible biological explanation for the beneficial effects reported across different pregnancy-related disorders and highlights Nrf2 as one of the most promising therapeutic targets for future translational and clinical research.

## 5. Clinical Evidence of Mushroom Consumption During Early Pregnancy

Evidence supporting the role of edible mushrooms in pregnancy-related metabolic and hypertensive outcomes has emerged from both experimental and human studies; however, the overall body of literature remains limited and heterogeneous, particularly with respect to dosage, study design, and translational relevance.

### 5.1. Preclinical Studies

Preclinical studies in animal models suggest that bioactive compounds derived from edible mushrooms, particularly β-glucans and phenolic fractions, may exert beneficial effects on glucose metabolism, inflammatory signaling, and oxidative stress regulation [108,129,130,131,132,133,134,135,136,137,138,139]. These mechanisms are highly relevant to the pathophysiology of GDM and hypertensive disorders of pregnancy, including PE.

Across preclinical studies conducted over the past decade, edible and medicinal mushrooms have predominantly been investigated as whole fruiting bodies (fresh, dried, or lyophilized powders), with fewer studies using standardized extracts or fermented preparations (Table 2). Also, we have presented the antidiabetic/metabolic effects, mechanisms, and safety profile of selected edible and medicinal mushrooms in non-pregnant experimental models (Table 3). The main bioactive constituents consistently implicated include β-glucans, phenolic compounds, and other antioxidant-related fractions. Experimental work has been largely based on pregnant rodent models (Wistar, Sprague-Dawley, BALB/c, ICR, and C57BL/6J strains), allowing relatively comparable assessment of metabolic, reproductive, and developmental outcomes in conditions relevant to gestational complications such as GDM, oxidative stress-related PE models, nutritional imbalance, and toxicant exposure.

Gestational exposure windows vary from early pregnancy (GD1–19 or GD1–21), encompassing implantation and organogenesis, to targeted pre- and post-implantation interventions, as well as perinatal and transgenerational designs extending through lactation and early postnatal life. Reported doses generally range from 100 to 600 mg/kg/day for oral gavage administration, whereas dietary supplementation studies employ inclusion levels of 1–10% in feed, reflecting both pharmacological and functional food-oriented approaches (Table 2).

At the maternal level, the most consistent effects include improvements in lipid metabolism (reduced triglycerides and cholesterol fractions), enhanced glycemic regulation in GDM models, and attenuation of liver enzyme alterations and oxidative stress biomarkers. In toxicant exposure models (e.g., lead), mushrooms demonstrate partial detoxifying and cytoprotective effects, including improved hematological and biochemical profiles and reduced oxidative damage, whereas in nutritional and metabolic programming models, they contribute to improved insulin sensitivity and energy metabolism regulation.

Fetal and offspring outcomes are generally neutral to beneficial at lower doses, with reports of improved fetal morphometry, reduced post-implantation loss, and better metabolic profiles in offspring. However, some dose-dependent adverse developmental effects have been observed at higher exposures (e.g., *A. brasiliensis* at 600 mg/kg/day), particularly affecting skeletal ossification, indicating a threshold beyond which developmental safety may be compromised [136]. Overall, the evidence suggests that mushrooms exert predominantly protective effects in gestational models, mediated mainly through antioxidant, anti-inflammatory, and metabolic regulatory mechanisms, while highlighting the importance of dose and exposure timing in determining safety and efficacy outcomes.

The studies summarized in Table 3 indicate that mushroom species have been extensively evaluated in non-pregnant in vivo models, predominantly in diabetic, hyperlipidemic, and metabolically challenged rodents. Across a wide range of doses, treatment durations, and mushroom preparations, these species consistently demonstrated beneficial metabolic effects, including improved glycemic control, lipid metabolism, antioxidant status, and pancreatic function, while adverse effects were rarely reported. Importantly, the available non-pregnant animal studies generally classified these mushrooms as well-tolerated and safe at the tested doses, supporting their favorable safety profile, although direct extrapolation to pregnancy remains limited due to the lack of pregnancy-specific studies.

However, despite these promising findings, animal studies are generally limited by differences in metabolic rate, immune responses, and placental physiology compared to humans. In addition, dosing regimens are often supra-physiological and not directly comparable to dietary intake levels in humans, which limit direct extrapolation to clinical settings.

### 5.2. Human Studies

In human populations, evidence on the role of mushroom consumption during pregnancy is predominantly derived from observational cohort studies, with only a limited number of interventional or controlled clinical trials available. A large cohort study reported that daily consumption of approximately 100 g of white button mushrooms during pregnancy was associated with a significantly lower incidence of gestational hypertension, PE, excessive gestational weight gain, and gestational diabetes mellitus compared with non-consumers [112]. These findings support a potential protective association between maternal mushroom intake and key adverse pregnancy outcomes, particularly metabolic and hypertensive disorders such as GDM and PE.

Evidence from biomarker-based clinical cohort data suggests a potential mechanistic link between mushroom-derived bioactive compounds and reduced risk of PE. Kenny et al. [155], within the Screening for Endpoints in Pregnancy (SCOPE) cohort, demonstrated that higher early-pregnancy plasma levels of ergothioneine, a dietary antioxidant predominantly obtained from mushrooms, were strongly associated with a markedly reduced incidence of PE. Using a threshold at the 90th percentile of the control distribution (≥462 ng/mL), only 1% of women above this level developed PE compared with 24.2% of those below the threshold, suggesting a potentially protective effect of elevated ergothioneine status against both pre-term and term PE. These findings, supported by prior experimental data in animal models of reduced uterine perfusion, indicate that ergothioneine may represent a biologically plausible mediator of the observed association between mushroom intake and reduced PE risk, although interventional confirmation in humans is still needed.

In addition, evidence from dietary pattern-based human studies further supports a potential beneficial role of mushroom-containing diets in maternal nutritional status. Nguyen et al. [156] reported that in pregnant women with pre-pregnancy overweightness or obesity, a dietary pattern characterized by mushrooms, roots, and dairy was positively associated with improved folate status (β = 0.052; 95% CI: 0.008–0.119; *p* < 0.05), suggesting a role of mushroom-containing dietary patterns in supporting micronutrient homeostasis during pregnancy. This was observed alongside broader associations between dietary patterns and serum iron, ferritin, and vitamin B12 levels, highlighting the relevance of mushrooms within nutritionally balanced dietary profiles in high-risk pregnant populations [156]. Complementary findings from another observational study further indicate that maternal diet during late pregnancy is associated with oxidative stress markers and stress-related endocrine parameters, including malondialdehyde (MDA) and cortisol levels in maternal and cord blood, suggesting that dietary quality, including nutrient-dense foods such as mushrooms, may influence maternal oxidative stress balance and fetal exposure to stress-related hormonal signals [157].

In a cohort of 449 pregnant women, factor analysis identified a “mushroom-vegetable-offal” dietary pattern among several distinct dietary profiles. Although not directly tested as a standalone protective factor, the overall dietary pattern analysis suggested that healthier dietary patterns, including those characterized by higher intake of mushrooms and plant-based foods, were associated with a reduced likelihood of developing GDM compared with a potato-cereal-based pattern (OR = 0.093, 95% CI: 0.025–0.342) [158].

Further evidence from a large cluster-based randomized controlled trial in 987 pregnant women in rural China identified five major dietary patterns (vegetable, meat, fruit, snack, and wheat staple patterns) and demonstrated that adherence to a vegetable dietary pattern was significantly associated with a reduced risk of PE (adjusted RR = 0.20, 95% CI: 0.04–0.98) and proteinuria (adjusted RR = 0.44, 95% CI: 0.24–0.80), suggesting a protective role of plant-rich dietary patterns in hypertensive pregnancy disorders (NCT02537392) [159]. Although mushrooms were not analyzed separately in this study, they are typically embedded within vegetable-rich dietary patterns, reinforcing their potential contribution to dietary profiles associated with lower PE risk.

In large population-based cohort studies, such as the Norwegian Mother and Child Cohort Study (MoBa), dietary patterns during pregnancy have been shown to influence the risk of PE, with overall food combinations appearing more important than single nutrients. In a cohort of over 23,000 nulliparous women, a dietary pattern characterized by high intake of vegetables, plant-based foods, and vegetable oils was associated with a significantly reduced risk of PE, whereas a pattern rich in processed foods, salty snacks, and sugary beverages was associated with increased risk. These findings are particularly relevant in the context of functional foods, including edible and medicinal mushrooms, which are typically classified within plant-based dietary patterns and are rich in bioactive compounds such as polysaccharides, dietary fiber, phenolics, and antioxidant molecules. This supports the concept that the potential effects of mushrooms in pregnancy are likely mediated not only through isolated bioactive compounds, but also through their role within broader healthy dietary patterns [160].

In addition to studies primarily addressing metabolic pregnancy outcomes such as GDM and hypertensive disorders, including PE, emerging clinical evidence suggests that maternal mushroom consumption may exert broader beneficial effects on pregnancy-related and offspring health outcomes. In a prebirth cohort study from Japan involving 1199 mother-child pairs, higher maternal mushroom intake during pregnancy was associated with a significantly lower risk of adverse behavioral outcomes in children, including reduced odds of peer problems and improved prosocial behavior at 5 years of age, suggesting potential long-term neurodevelopmental benefits of maternal dietary mushroom intake beyond metabolic regulation [161]. Complementary evidence from a cross-sectional study in pregnant women further indicates that dietary patterns containing mushrooms may play a role in maternal nutritional status, as “mushrooms, roots, and dairy”-based dietary patterns were found to mediate the relationship between serum vitamin D levels and iron status, highlighting a potential contribution of mushroom intake to erythropoiesis-related micronutrient balance during pregnancy [162]. Such findings are particularly relevant in the context of pregnancy-associated anemia and metabolic adaptation. Overall, although direct clinical evidence linking mushrooms to reduced risk of GDM or PE remains limited, these studies support a broader nutritional and functional role of mushrooms in pregnancy, spanning micronutrient homeostasis and child developmental outcomes [161,162]. These findings are relevant in the context of functional foods, including edible and medicinal mushrooms, which are typically consumed within mixed plant-based dietary patterns rather than as isolated dietary exposures in epidemiological studies.

Furthermore, clinical and epidemiological evidence on mushroom consumption during pregnancy indicates that socio-cultural factors may also significantly influence maternal dietary intake. A qualitative study conducted in the Acholi subregion of Northern Uganda highlighted the presence of multiple food taboos during pregnancy that may contribute to maternal undernutrition in rural settings [163]. Among the foods identified as culturally restricted were offals, meat products, fruits, legumes, honey, and notably mushrooms, which were explicitly considered taboo foods in some communities. These restrictions were primarily driven by cultural beliefs, societal norms, and traditional perceptions regarding pregnancy outcomes. The study emphasizes that such dietary prohibitions may limit food diversity and nutrient intake during pregnancy, potentially exacerbating the risk of maternal undernutrition and associated adverse pregnancy outcomes in already vulnerable populations [163].

## 6. Safety Considerations and Dietary Recommendations in Pregnancy

Edible mushrooms have long been recognized as safe and nutritious foods and are increasingly being investigated for their potential role in preventing pregnancy-related metabolic and hypertensive disorders [112,164]. Their favorable nutritional profile, characterized by low energy density, high fiber content, essential micronutrients, and a wide range of bioactive compounds, supports their inclusion in a balanced maternal diet. Nevertheless, several safety and regulatory considerations should be addressed before mushroom-based nutritional strategies can be widely recommended for pregnancy-specific health outcomes.

The safety profile of commonly consumed edible mushrooms, such as *P. ostreatus* and *A. bisporus,* is generally well established when they are obtained from reliable sources and adequately cooked before consumption. These species provide nutrients and bioactive compounds, including ergothioneine, β-glucans, dietary fiber, selenium, and vitamins, without exposing consumers to pharmacologically concentrated doses. Proper thermal processing reduces microbial contamination and improves digestibility, while minimizing the risk associated with naturally occurring toxic compounds present in some species [165,166]. However, mushrooms are known bioaccumulators and may concentrate heavy metals, pesticides, and environmental pollutants depending on cultivation conditions and geographical origin. Hence, the presence of pesticide residues cannot be entirely excluded and may vary according to cultivation practices and regulatory compliance. Sourcing mushrooms from certified producers and controlled cultivation systems represents an important additional precautionary measure for reducing maternal exposure to environmental contaminants during pregnancy. Consequently, product quality and source traceability remain important considerations, particularly during pregnancy, when maternal exposure to contaminants may affect fetal development [166].

In contrast to whole edible mushrooms, the safety of medicinal mushroom extracts and dietary supplements during pregnancy remains insufficiently characterized. Commercial preparations derived from species such as *G. lucidum*, *L. edodes*, *H. erinaceus*, and *G. frondosa* are increasingly marketed for their antioxidant, immunomodulatory, and metabolic benefits [167]. However, most available evidence originates from in vitro studies, animal models, or non-pregnant populations. Given the limited number of pregnancy-specific preclinical and clinical studies, the effects of concentrated mushroom extracts on maternal physiology, placental function, fetal development, and potential interactions with medications remain largely unknown. Therefore, routine use of medicinal mushroom supplements during pregnancy cannot currently be recommended without medical supervision and further clinical validation.

Considering the central role of oxidative stress, endothelial dysfunction, and chronic low-grade inflammation in the pathogenesis of both PE and GDM, ergothioneine may represent one of the key mechanistic links between mushroom consumption and improved pregnancy outcomes. Nevertheless, direct clinical evidence evaluating ergothioneine intake, circulating concentrations, and pregnancy-related endpoints remains scarce. Further prospective studies and randomized controlled trials are required to determine optimal intake levels and establish causality. Potential interactions with medications should also be considered. Experimental evidence suggests that certain mushroom-derived compounds may influence glucose metabolism, blood pressure regulation, coagulation pathways, and immune responses [168]. Consequently, theoretical interactions with antidiabetic, antihypertensive, anticoagulant, or immunomodulatory therapies cannot be excluded.

Based on the currently available evidence, the inclusion of edible mushrooms as part of a healthy and diversified diet appears to be a safe and potentially beneficial nutritional practice during pregnancy. However, specific dietary recommendations regarding mushroom intake for the prevention of PE or GDM cannot yet be established.

## 7. Limitations of Current Clinical Evidence

Mushrooms are increasingly consumed as foods and dietary supplements, and substantial experimental evidence highlights the antidiabetic and antioxidant potential of mushroom-derived polysaccharides, terpenoids, phenolic compounds, and ergothioneine [115]. Numerous preclinical studies have demonstrated beneficial effects of these bioactive metabolites on glucose homeostasis, insulin sensitivity, oxidative stress, inflammation, and endothelial function [117,133,134,135,137]. However, despite these promising findings, clinical evidence remains limited and occasionally inconsistent.

Current human studies are characterized by several important limitations. Most investigations rely on whole mushrooms or crude mushroom extracts rather than purified bioactive compounds and are conducted over relatively short intervention periods [169]. In addition, the available evidence is predominantly observational, limiting the ability to establish causal relationships between mushroom consumption and improved metabolic outcomes during pregnancy. Comparisons across studies are further complicated by considerable heterogeneity in mushroom species, cultivation conditions, processing methods, dietary patterns, baseline nutritional status, and potential lifestyle-related confounders.

Moreover, standardized information regarding timing of exposure during pregnancy, precise intake quantification, dose–response relationships, bioavailability, placental transfer, and the contribution of individual bioactive compounds remains insufficient. Although ergothioneine has emerged as a particularly promising mushroom-derived antioxidant, human evidence regarding its specific role in preventing or mitigating PE and GDM is still scarce. The lack of mechanistic clinical validation further limits the translation of epidemiological observations into evidence-based dietary recommendations.

Additional challenges arise from the limited number of studies conducted specifically in pregnant populations. As a result, much of the current understanding relies on extrapolation from experimental models or studies performed in non-pregnant individuals. Consequently, the efficacy, optimal dosage, long-term safety, and potential interactions of mushroom-derived bioactive compounds during pregnancy remain incompletely understood. Another limitation of the available evidence is the insufficient reporting and control of potential confounding factors such as overall diet, lifestyle habits, and exposure to environmental pollutants, which may influence the observed outcomes. Furthermore, the current body of evidence lacks standardized reporting of study quality, and few studies apply formal risk-of-bias or evidence-grading frameworks, which further limits the comparability and strength of conclusions across studies.

## 8. Future Perspectives and Research Directions

Current evidence suggests that edible and medicinal mushrooms may contribute to the prevention and management of pregnancy-related metabolic and hypertensive disorders, including GDM and PE. Nevertheless, substantial research gaps remain, warranting further investigation before definitive clinical recommendations can be established.

Future studies should focus on identifying the mushroom species and bioactive metabolites that exert the strongest protective effects during pregnancy. Particular attention should be directed toward ergothioneine, β-glucans, phenolic compounds, terpenoids, and other emerging metabolites due to their antioxidant, anti-inflammatory, immunomodulatory, and metabolic activities. Among these compounds, ergothioneine appears especially promising because of its potent redox-regulating properties, selective accumulation in tissues exposed to oxidative stress through the SLC22A4 (OCTN1) transporter, and its potential role in protecting placental and endothelial function.

Large prospective cohort studies and well-designed randomized controlled trials are needed to determine whether mushroom consumption or supplementation with specific mushroom-derived compounds can effectively reduce the incidence or severity of PE and GDM. Future clinical investigations should establish optimal intake levels, critical exposure windows during pregnancy, dose–response relationships, bioavailability, placental transfer, and long-term maternal and offspring outcomes.

Mechanistic studies should further explore the effects of mushroom-derived metabolites on trophoblast invasion, placental angiogenesis, endothelial dysfunction, mitochondrial homeostasis, oxidative stress signaling, immune tolerance, gut microbiota modulation, and glucose metabolism. Particular emphasis should be placed on pathways implicated in PE and GDM pathogenesis, including Nrf2/KEAP1 signaling, inflammatory cytokine networks, insulin signaling pathways, and vascular regulatory mechanisms.

Advances in metabolomics, nutrigenomics, microbiome research, and biomarker-based approaches may facilitate the identification of molecular signatures associated with beneficial responses to mushroom consumption. In addition, circulating ergothioneine concentrations may represent a promising biomarker for assessing antioxidant status and evaluating the efficacy of mushroom-based nutritional interventions during pregnancy.

Ultimately, the integration of nutritional, clinical, and mechanistic research may support the development of evidence-based dietary strategies utilizing edible and medicinal mushrooms as functional foods to improve maternal and fetal health outcomes during early pregnancy.

## 9. Literature Search Strategy

This study is designed as a narrative review of the current literature on edible and medicinal mushrooms and their potential relevance to maternal metabolic health, with a focus on oxidative stress, inflammation, and pregnancy-related metabolic disorders. The narrative approach was selected due to the high heterogeneity of available evidence, which includes mechanistic in vitro studies, animal models, observational studies, and a limited number of clinical investigations, thereby precluding systematic or meta-analytic synthesis.

A comprehensive literature search was conducted using the following electronic databases: PubMed/MEDLINE, Scopus, Web of Science Core Collection, ScienceDirect, and Google Scholar. The search included studies published from January 2006 to March 2026, with particular emphasis on publications from the last decade due to the growing interest in mushroom-derived bioactive compounds and their potential implications for maternal health. Only articles published in English were considered. Additional relevant studies were identified through manual screening of the reference lists of selected publications to ensure inclusion of key foundational and highly cited studies. The search strategy combined controlled vocabulary (MeSH terms where applicable) and free-text keywords related to edible and medicinal mushrooms, pregnancy, oxidative stress, inflammation, and metabolic disorders. Keywords included combinations of “edible mushrooms,” “medicinal mushrooms,” “functional mushrooms,” “ergothioneine,” “β-glucans,” “polysaccharides,” “phenolic compounds,” “terpenoids,” “pregnancy,” “maternal health,” “placenta,” “early pregnancy,” “preeclampsia,” “gestational diabetes mellitus,” “pre-gestational diabetes,” “oxidative stress,” “redox balance,” “antioxidant activity,” “inflammation,” “immune modulation,” and “endothelial dysfunction,” linked using Boolean operators (AND, OR).

Eligible publications included original research articles (in vitro and in vivo), clinical trials, animal experiments, systematic reviews, meta-analyses, and relevant narrative reviews. Studies were included if they investigated edible or medicinal mushrooms, mushroom extracts, or isolated bioactive compounds in relation to metabolic regulation, oxidative stress, inflammation, or pregnancy-related metabolic complications. Studies addressing mechanisms associated with PE, GDM, pre-gestational diabetes, placental dysfunction, endothelial injury, oxidative stress, inflammation, glucose homeostasis, and maternal–fetal health were prioritized. Publications not written in English, conference abstracts, editorials, letters, patents, unpublished data, duplicate records, and studies lacking sufficient methodological information or relevance to the topic were excluded.

The selection process involved an initial screening of titles and abstracts, followed by a full-text evaluation of potentially relevant articles. Study inclusion was based on predefined relevance criteria (objective screening of topic relevance and study type), whereas the synthesis of the included evidence involved thematic organization and interpretative integration by the authors, reflecting the expert judgment typically applied in narrative reviews. Greater weight was given to systematic reviews and meta-analyses, well-controlled in vivo experimental studies and human clinical studies when available.

Preclinical studies were used primarily to support mechanistic insights, while clinical evidence was prioritized for translational relevance. Observational studies were used to complement clinical and experimental findings where applicable.

No formal risk-of-bias scoring system was applied; however, study design, sample size, and methodological rigor were considered qualitatively during interpretation.

## 10. Conclusions

PE and GDM remain among the most prevalent pregnancy-related complications, contributing substantially to maternal and fetal morbidity worldwide. Accumulating evidence indicates that oxidative stress, chronic inflammation, endothelial dysfunction, and metabolic disturbances play central roles in their pathogenesis, particularly during early placental development. In this context, edible and medicinal mushrooms have emerged as promising functional foods owing to their rich nutritional profile and diverse repertoire of bioactive metabolites.

Mushroom-derived compounds, including ergothioneine, β-glucans, phenolic compounds, terpenoids, dietary fibers, and vitamin D_2_, exhibit antioxidant, anti-inflammatory, immunomodulatory, and metabolic regulatory activities that may target several pathways implicated in PE and GDM. Experimental studies suggest that these metabolites can improve redox homeostasis, support glucose metabolism, modulate immune responses, preserve endothelial function, and protect placental tissues from oxidative injury. Among them, ergothioneine has attracted particular interest due to its potent antioxidant properties, tissue-specific accumulation through the OCTN1 transporter, and potential role in protecting maternal vascular and placental health.

Therefore, current evidence supports biological plausibility, but not yet clinical recommendation, for mushroom consumption or mushroom-derived compounds as preventive or therapeutic strategies for PE and GDM. Future research should focus on addressing several key knowledge gaps that currently limit the translational potential of edible and medicinal mushrooms in pregnancy-related metabolic disorders. These include the lack of well-designed randomized controlled trials in pregnant populations, insufficient data on bioavailability, dose–response relationships, timing of exposure during gestation, and placental transfer of mushroom-derived bioactive compounds. In addition, standardized characterization of mushroom preparations and isolated compounds is needed to improve comparability across studies. Another important research priority is the establishment of robust evidence on safety, long-term maternal–fetal outcomes, and mechanistic validation in clinically relevant models. Overall, future studies should move beyond descriptive and mechanistic findings toward well-controlled clinical investigations to determine whether mushroom-derived compounds can be translated into evidence-based nutritional strategies in pregnancy.

## Figures and Tables

**Table 1:** Selected edible and medicinal mushrooms, their major bioactive and antioxidant compounds, potential relevance to maternal health during pregnancy, and proposed mechanisms associated with oxidative stress, inflammation, and metabolic dysregulation.

Mushroom Species	Major Bioactive Compounds	Major Antioxidant Compounds	Potential Relevance to PE and GDM	Proposed Mechanism	References
*Pleurotus ostreatus*	β-Glucans, dietary fiber, ergothioneine, phenolic compounds	Ergothioneine, gallic acid, protocatechuic acid	Support of glucose metabolism; antioxidant protection	β-Glucan-mediated improvement of insulin sensitivity; ROS scavenging; modulation of inflammatory cytokines	[25,74,75,76,77]
*Agaricus bisporus*	Ergothioneine, selenium, phenolic compounds	Ergothioneine, phenolic acids	Reduction in oxidative stress; support of maternal micronutrient intake	Ergothioneine-mediated antioxidant activity; selenium-dependent antioxidant enzymes	[78,79,80,81]
*Lentinula edodes*	Lentinan, β-glucans, phenolic compounds	Lentinan, phenolic compounds	Immune modulation and anti-inflammatory potential	β-Glucan-induced immune regulation; suppression of pro-inflammatory mediators	[81,82,83,84]
*Ganoderma lucidum*	Triterpenoids, polysaccharides, ganoderic acids	Ganoderic acids, polysaccharides, polyphenolics	Anti-inflammatory and antioxidant activity	Triterpenoid-mediated inhibition of oxidative stress and inflammatory pathways	[22,26,83,85,86]
*Hericium erinaceus*	Erinacines, hericenones, polysaccharides, phenolics	Erinacines, hericenones, phenolics	Neuroprotective and antioxidant potential	Enhancement of antioxidant defense systems; modulation of neurotrophic factors	[83,87,88,89,90,91]
*Flammulina velutipes*	Ergothioneine, polysaccharides, phenolic compounds	Ergothioneine, phenolics	Support of antioxidant defense and metabolic balance	Ergothioneine- and phenolic-mediated free radical scavenging	[83,92,93,94]

**Table 2:** Preclinical pregnancy, GDM, PE and developmental toxicity related studies of edible and medicinal mushrooms.

Species	Mushroom Form	Experimental Model	Gestational Exposure	Dose/Route	Maternal Outcomes	Fetal/Offspring Outcomes	Mechanisms	Safety	Ref
*L. edodes*	Whole culinary mushroom	Pregnant rats	GD1–19 (pre-implantation exposure) and GD9–19 (post-implantation exposure)	Dietary intake	↓ triglycerides; no maternal toxicity	No fetal morphological changes	Hypolipidemic effects	Safe	[129]
*L. edodes*	Mycelialextract (AHCC)	Pregnant Balb/c mice and offspring	GD14 to weaning (PND21); offspring exposed in utero and during early postnatal life	AHCC 4 g/kg BW/day and/or penicillin V 31 mg/kg BW/day via drinking water	Gut microbiota modulation; ↓ cytokines; ↓ NF-κB	Improved offspring microbiota; ↑ SCFA bacteria	Microbiota-immune modulation	Safe	[130]
*L. edodes*	AHCC	Pregnant ICR mice (multi-generational design)	GD3 and GD5 (pre-exposure), radiation on GD9; fetuses analyzed GD18	AHCC 0.2 mg/g BW (2% solution) intraperitoneally prior to 1.4 Gy γ-irradiation			Maternal immune modulation and radioprotective effects during organogenesis	Safe	[131]
*L. edodes*	Fermented (FLE) mushroom	Pregnant C57BL/6J mice under high-fat diet (overnutrition model)	Pregnancy and lactation period	Dietary supplementation of FLE at 1%, 3%, and 5% in high-fat diet	Reduced serum insulin levels and HOMA-IR; improved maternal insulin sensitivity; improved lipid metabolism and reduced fat deposition in dams; activation of hepatic PI3K/AKT signaling	Reduced offspring body fat percentage and visceral fat; improved metabolic profile at weaning (↓ glucose, ↓ insulin, ↓ HOMA-IR); increased weaning litter weight; activation of hepatic PI3K/AKT pathway and upregulation of lipolytic genes (ATGL, HSL, CPT1)	Modulation of PI3K/AKT signaling pathway; improved lipid oxidation and lipolysis; enhanced insulin signaling	Safe	[132]
*L. edodes*	Lyophilized powder	Streptozotocin-induced GDM-in pregnant Wistar rats	Before implantation (GD1–19) and after implantation (GD9–19)	100 mg/kg/day orally	Increased maternal insulin levels despite persistent hyperglycemia; reduced ALT, AST, triglycerides, and total cholesterol; reversal of STZ-induced hepatic damage; improved oxidative stress parameters	Reduced post-implantation losses and improved conceptus viability; no major fetal morphological toxicity reported	Antioxidant effects	Safe	[108]
*L. edodes*	Lyophilized whole mushroom	Streptozotocin-induced GDM in pregnant rats	GD1–19 (continuous gestational exposure; GDM induced GD4)	100 or 200 mg/kg/day by oral administration (gavage)	Reduced plasma glucose, urea, triglycerides, and AST; modulation of antioxidant enzyme activity (↓catalase in plasma reported); partial improvement of metabolic and biochemical parameters	Reduced preimplantation loss compared with diabetic control; no full reversal of STZ-induced pregnancy damage throughout gestation	Antioxidant and metabolic modulation effects; partial protection against hyperglycemia-induced oxidative and metabolic stress	No toxicity	[138]
*L. edodes*	Lyophilized mushroom powder	Sprague–Dawley rats (pregnant dams and offspring; preclinical developmental nutrition model)	From gestation day 4 through postnatal day 126 (including gestation, lactation, and postnatal development)	Dietary incorporation (0%, 1%, 4%, 10% *w*/*w* in feed; ad libitum consumption)	Reduced serum total cholesterol, HDL, non-HDL cholesterol, and triglycerides; dose-dependent improvement in lipid profile; modulation of serum metabolic biomarkers	Reduced body weight in male offspring at higher doses (4% and 10%); improved serum lipid and metabolic parameters; sex-dependent effects on glucose/insulin regulation	Modulation of lipid metabolism; antioxidant-related activity (ORAC-based lipophilic/hydrophilic capacity); regulation of insulin and leptin signaling pathways	Generally safe	[139]
*P. ostreatus*	Aqueous extract	Transgenerational rat model	Maternal exposure during gestation and lactation (sucrose consumption); offspring intervention in adulthood (post-weaning period)	Maternal: 5% sucrose solution in drinking water; Offspring: aqueous mushroom extract administered after 3 months of sucrose exposure (duration: 1 month)	No direct maternal metabolic outcomes	Offspring showed increased adiposity due to sucrose exposure despite unchanged body weight; mushroom extract reduced plasma cholesterol and triglycerides, decreased total adiposity, and reduced visceral adipocyte size	Antihyperlipidemic and anti-obesity effects	Safe	[140]
*A. bisporus*	Dried mushroom powder (“mushroom flour”); rich in β-glucans and antioxidant compounds	Pregnant Wistar rats exposed to lead (Pb) toxicity	GD1–19	100 mg/kg/day by oral gavage; Pb 100 mg/L in drinking water	Pb exposure significantly increased Pb accumulation in maternal blood, placenta, liver, kidney, and bone; co-administration with A. bisporus significantly reduced Pb levels in blood, placenta, and liver, partially reducing renal and bone Pb accumulation	Reduced fetal brain Pb accumulation in co-exposed group compared with Pb-only group, suggesting partial fetal protection against transplacental Pb transfer	Chelating activity reduction of Pb absorption, distribution, and oxidative damage	Safe	[135]
*A. bisporus*	Suspension	Pregnant Wistar rats co-exposed to lead (Pb) toxicity	GD1–19	A. bisporus 100 mg/kg/day by oral gavage; Pb 100 mg/L in drinking water	Pb exposure reduced maternal weight gain and impaired hematological, biochemical, and oxidative stress parameters	Partial recovery of fetal morphological and skeletal abnormalities induced by Pb exposure	Antioxidant, metal-chelating, and cytoprotective effects	Safe	[134]
*Agaricus brasiliensis*	Whole mushroom	Streptozotocin -induced GDM rat model	Perinatal exposure: before and after GDM induction (Abb and Aba protocols)	Daily oral intake of mushroom (dietary supplementation; experimental perinatal administration)	Improved glycemic control (reduced hyperglycemia prior to STZ and stabilization after induction); improved lipid profile (↓ triglycerides, ↓ cholesterol, ↑ HDL); reduced liver enzyme markers (ALT, AST); improved oxidative stress status	Protection against STZ-induced embryofetal damage; reduced external abnormalities in conceptus; improved overall embryofetal development parameters	Antioxidant activity, reduction in oxidative stress, modulation of glucose and lipid metabolism, β-glucan–related metabolic effects	Safe	[133]
*A. brasiliensis*	Powder-dehydrated reconstituted whole mushroom	Pregnant Wistar rats	Entire gestational period (GD1–21)	300 or 600 mg/kg/day by oral gavage	Maternal fertility and body weight were monitored; no major maternal reproductive toxicity reported	Increased sternebrae agenesis at 600 mg/kg/day; incomplete sternebrae ossification observed at both 300 and 600 mg/kg/day; offspring evaluated for vitality, morphology, physical and neurobehavioral development	Possible developmental toxicity at high doses affecting fetal skeletal organogenesis	Low/moderate toxicity at high dose	[136]
*G. lucidum*	Whole mushroom powder	Streptozotocin-induced GDM in pregnant Wistar rats	Before implantation (GD1–19) and after implantation (GD9–19)	100 mg/kg/day by oral gavage	Improved glucose tolerance (reduced OGTT response); decreased AST and ALT	Improved fetal morphometry (increased head, thorax, craniocaudal and tail measurements)	Antioxidant and antihyperglycemic activity	Safe	[137]

**Table 3:** Biological activities, antidiabetic/metabolic effects, mechanisms, and safety profile of selected edible and medicinal mushrooms in non-pregnant experimental models.

Species	Mushroom Form	Model	Exposure	Dose/Route	Main Outcomes	Mechanisms	Safety	Ref
*L. edodes*	Methanolic extract (DMRO-34, DMRO-356 strains)	In vitro and in vivo toxicity (HeLa, MCF-7; Wistar rats)	Acute and subacute (14–28 days)	Up to 5000 mg/kg oral	No observed adverse effects (NOAEL); well tolerated in rats; strain-dependent cytotoxicity in cancer cell lines	β-glucan–associated bioactivity; selective in vitro cytotoxicity	Safe at tested doses; no in vivo toxicity observed	[141]
*L. edodes*	Cultured mycelial extract	Wistar rats (male and female)	28-day repeated exposure (OECD 407)	2000 mg/kg/day, oral gavage	No mortality or clinical toxicity; slight transient reduction in body weight and food intake (mainly males); no consistent hematological or biochemical toxicity	No significant organ weight changes; no histopathological lesions attributable to treatment; NOAEL > 2000 mg/kg/day	Safe under repeated oral exposure; no observed adverse effects at tested dose	[142]
*L. edodes*	Whole powsered mushroom (dietary supplementation)	Male Wistar rats (HFD-induced metabolic dysfunction model)	30-day oral exposure	100 mg/kg/day, oral gavage	Normal diet parameters restored under HFD: reduced total cholesterol, triglycerides, glucose, urea, AST/ALT; decreased lipid peroxidation (MDA); improved oxidative stress profile	No toxic effects observed; improvement of metabolic and hepatic biomarkers under HFD conditions	Safe at nutritionally relevant dose with hypolipidemic, hypoglycemic and antioxidant effects	[143]
*P. ostreatus* and *Lentinus subnudus*	Powdered dietary supplementation	STZ-induced diabetic model (Wistar rats)	14 days	10–20% diet inclusion	↓ α-amylase, α-glucosidase, ACE; ↑ NO; improved metabolic profile	Enzyme inhibition, antioxidant activity and metabolic regulation	No toxicity reported	[144]
*P. ostreatus*	Total fungal polysaccharides	Type 2 diabetes rat model (HFD + STZ)	4-week treatment after induction of diabetes	100, 200, 400 mg/kg BW oral gavage	↓ fasting blood glucose; ↓ hyperlipidemia (TC, TG, LDL-C); ↑ insulin sensitivity; ↑ liver and muscle glycogen; improved metabolic profile; ↓ oxidative stress (↓ MDA); ↑ antioxidant enzymes (SOD, CAT, GSH-Px)	Activation of insulin signaling and glucose metabolism pathways; improved antioxidant defense system; enhanced glycogen synthesis; reduced lipid peroxidation	No toxicity reported	[75]
*A. bisporus* and *P. ostreatus*	Mixed dried powder diet (fruiting bodies)	STZ-induced diabetic rats + diet-induced hypercholesterolemia (Sprague Dawley rats)	3–6 weeks dietary intervention	15 and 30 g/day in feed (oral dietary supplementation)	↓ blood glucose; reversal of hyperglycemia; ↓ AST, ALT, ALP; improved lipid profile (↓ hyperlipidemia, ↑ HDL); normalization of serum electrolytes (Na^+^, K^+^, HCO_3_^−^, Cl^−^); ↑ total protein; protection of liver and kidney function; improved metabolic status	Antioxidant and hepatoprotective effects; modulation of lipid metabolism; improved glucose homeostasis; likely reduction in oxidative stress and tissue damage	No toxicity reported	[145]
*A. bisporus* (*ABP*); *Pleurotus eryngii*; *Boletus* spp.; *L. edodes*; *P. ostreatus*	Purified fungal polysaccharides (multiple edible mushrooms)	Type 2 diabetes mouse model (HFD + multiple low-dose STZ in C57BL/6J mice)	Chronic treatment during diabetic induction period (after HFD + STZ)	200 mg/kg BW oral gavage daily (metformin 70 mg/kg as reference)	All polysaccharides improved diabetic phenotype; ABP strongest: ↓ fasting glucose; improved glucose tolerance (GTT) and insulin sensitivity (ITT); ↓ hyperphagia/polydipsia; ↑ body weight; ↓ TG, TC, LDL-C; ↑ HDL-C; ↓ ALT/AST; improved liver index; ↓ hepatic steatosis and inflammation; protection of pancreatic islets	Activation of insulin signaling (IRS1/PI3K/Akt pathway); activation of AMPK energy-sensing pathway; improved lipid metabolism; metabolomics showed reversal of diabetes-related disturbances	No toxicity reported	[146]
*A. bisporus*	Hot water extract; ethanol extract (ABEE); methanol extract (ABME) from fruiting body	STZ-induced diabetic rats (Sprague Dawley) and alloxan-induced diabetic rats (Wistar/Albino rats)	7 days (hot water extract) and 30 days (ABEE/ABME)	100–400 mg/kg bw/day (hot water); 200 mg/kg bw/day oral (ABEE/ABME)	↓ blood glucose; ↑ insulin; improved glycemic curve; ↑ liver glycogen; ↑ protein; ↓ TC, TG, PL; ↓ AST/ALT/ALP/LDH; ↓ oxidative stress markers (MDA, TBARS); improved pancreatic histology (β-cell repopulation); improved metabolic profile	Antioxidant activity (↑ SOD, CAT); anti-lipid peroxidation; β-cell protection/regeneration; improved lipid metabolism; improved insulin response	No toxicity reported	[147]
*A. bisporus*	Hot water extract	In vivo (Sprague Dawley rats; streptozotocin-induced diabetes)	7 days	0, 100, 200, 400 mg/kg bw/day; oral	Dose-dependent improvement of diabetic symptoms; significant reduction in serum glucose (up to 29.68% at 400 mg/kg), increased insulin (up to 78.5%), improved antioxidant status (↑SOD, ↑catalase), decreased lipid peroxidation (↓MDA), histological regeneration of pancreatic Langerhans islets with increased β-cellularity	Antioxidant protection, β-cell regeneration/repopulation, reduced oxidative stress and lipid peroxidation	No toxicity reported	[148]
*A. brasiliensis*	Dried fruit body (dietary powder, 1% diet inclusion)	In vivo (Wistar rats; cholesterol-enriched diet model, non-diabetic)	32 days	1% *w*/*w* in AIN-93-based diet (±1% cholesterol)	Improved glycemic response (attenuated glucose curve), reduced total cholesterol and triglycerides, increased HDL, reduced hepatic lipid accumulation, increased fecal lipid excretion	High dietary fiber/polysaccharides reduce intestinal lipid absorption; increased fecal sterol excretion; modulation of lipid metabolism and hepatic deposition	No adverse effects reported	[149]
*G. lucidum* and *A. brasiliensis*	Whole mushroom preparations	STZ-induced diabetic rats (Wistar)	2 weeks	1 g/kg/day oral	↑ antioxidant enzyme activity in leukocytes; ↓ TBARS (lipid peroxidation); restored oxidative balance	Strong antioxidant effect (enhanced enzymatic defense against ROS)	No toxicity reported	[150]
*G. lucidum* and *A. brasiliensis*	Submerged culture mycelium powder	STZ-induced diabetic rats (Wistar)	2 weeks	1 g/kg/day oral	↓ blood glucose; ↓ HbA1c; ↑ erythrocyte count; improved RBC stability; ↑ erythropoiesis	Antioxidant and hematopoietic modulation (improved oxygen transport and RBC resilience)	No toxicity reported	[151]
*G. lucidum*	Low-molecular-weight polysaccharides (Gl-PS)	STZ-induced diabetic rats (Sprague-Dawley)	8 weeks	200 mg/kg oral (metformin 100 mg/kg comparator)	↓ fasting glucose, TG, cholesterol, NO; improved pancreatic histology; β-cell protection/regeneration	Anti-apoptotic effects (↑Bcl-2, PDX-1; ↓Bax, iNOS, Casp-3) and antioxidant enzyme activation	No toxicity reported	[152]
*G. lucidum*	Polysaccharides extract	STZ-induced diabetic rats	30 days	60, 120, 180 mg/kg (i.p./DMSO)	↓ blood glucose; ↑ serum insulin; ↓ lipid peroxidation; ↑ enzymic and non-enzymic antioxidants	Antioxidant action and improved insulin regulation	No toxicity reported; dose-dependent protective effects	[153]
*G. lucidum*	Polysaccharides (GLP)	HFD + STZ-induced T2DM rats	28–30 days (implied intervention period)	Oral administration	↓ fasting blood glucose; ↓ insulin; modulation of gut microbiota; improved metabolic profile	Gut microbiota remodeling and metabolomics shifts (amino acid, carbohydrate, lipid metabolism)	No toxicity reported	[154]

## Data Availability

No new datasets were generated or analyzed during the current review. All information presented in this manuscript is derived from previously published studies, which are cited accordingly.

## Abbreviations

The following abbreviations are used in this manuscript:

PE	Preeclampsia
GDM	Gestational diabetes mellitus
ROS	Reactive oxygen species
WHO	World health organization
Nrf2	Nuclear factor erythroid 2-related factor 2
